# Nociceptin Induces Hypophagia in the Perifornical and Lateral Hypothalamic Area

**DOI:** 10.1371/journal.pone.0045350

**Published:** 2012-09-17

**Authors:** Matthew P. Parsons, Julia Burt, Amanda Cranford, Christian Alberto, Katrin Zipperlen, Michiru Hirasawa

**Affiliations:** Division of Biomedical Sciences, Faculty of Medicine, Memorial University, St. John’s, Newfoundland and Labrador, Canada; Dalhousie University, Canada

## Abstract

Nociceptin/orphanin FQ (N/OFQ) is known to induce food intake when administered into the lateral ventricle or certain brain areas. This is somewhat contradictory to its reward-suppressing role, as food is a strong rewarding stimulus. This discrepancy may be due to the functional diversity of N/OFQ’s target brain areas. N/OFQ has been shown to inhibit orexin and melanin-concentrating hormone (MCH) neurons, both of which are appetite-inducing cells. As the expression of these neurons is largely confined to the lateral hypothalamus/perifornical area (LH/PFA), we hypothesized that N/OFQ inhibits food intake by acting in this area. To test this hypothesis, we examined the effect of local N/OFQ infusion within the LH/PFA on food intake in the rat and found that N/OFQ decreased sugar pellet as well as chow intake. This effect was not seen when the injection site was outside of the LH/PFA, suggesting a site-specific effect. Next, to determine a possible cellular mechanism of N/OFQ action on food intake, whole cell patch clamp recordings were performed on rat orexin neurons. As previously reported in mice, N/OFQ induced a strong and long lasting hyperpolarization. Pharmacological study indicated that N/OFQ directly inhibited orexin neurons by activating ATP-sensitive potassium (KATP) channels. This effect was partially but significantly attenuated by the inhibitors of PI3K, PKC and PKA, suggesting that the N/OFQ signaling is mediated by these protein kinases. In summary, our results demonstrate a KATP channel-dependent N/OFQ signaling and that N/OFQ is a site-specific anorexic peptide.

## Introduction

The rewarding nature of food can induce caloric intake in excess of our energy requirements by engaging the brain’s reward circuitry in a manner not unlike drugs of abuse [1]–[3]. Such excessive caloric intake is a major cause of obesity and its comorbidities. Given the clear link between overfeeding and obesity, understanding the neural underpinnings of reward-related feeding is paramount.

Endogenous opioids are known for their critical role in the brain’s reward system mediating the responses to substances of abuse [4] and palatable foods [5]. Nociceptin/orphanin FQ (N/OFQ) is an endogenous opioid known to bind the N/OFQ peptide (NOP) receptor [6] and to have anti-reward properties. Specifically, it functionally opposes the actions of other endogenous opioids on reward-related behavioural responding [7], [8] and decreases drug-induced dopamine release in the nucleus accumbens [9], [10]. Despite the rewarding nature of food, however, N/OFQ has been shown to induce, rather than inhibit, food intake when injected into the lateral ventricle [11] or several brain nuclei [12], [13]. This paradox of N/OFQ’s anti-reward and pro-feeding actions remains unresolved. A possible explanation is the functional heterogeneity of brain areas and neuronal types that mediate the N/OFQ effects.

The lateral hypothalamus/perifornical area (LH/PFA) is regarded as a critical brain region involved in motivated behaviours including feeding [3], [14]. There are two populations of peptidergic neurons that are expressed more or less exclusively in this region, i.e. orexin and melanin-concentrating hormone (MCH) neurons. Both of these neurons promote reward-related behavioural responses [15], [16] including palatable food intake [17], [18], suggesting that the reward-related functions of the LH/PFA can be largely attributed to these neurons. At the cellular level, N/OFQ induces a potent and reversible hyperpolarization of MCH neurons by activating G-protein dependent inwardly rectifying potassium (GIRK) channels [19]. Orexin neurons are also inhibited by N/OFQ via activation of potassium current [20], although the identity of the effector channel has yet to be identified. Based on these studies demonstrating that N/OFQ negatively regulates feeding neurons in the LH/PFA, we hypothesized that the LH/PFA is the site that N/OFQ would have an inhibitory effect on food intake.

Here, we tested the effect of intra-LH/PFA injection of N/OFQ on food intake. Furthermore, we investigated the cellular mechanism by which N/OFQ inhibits orexin neurons and identified KATP currents as being responsible for the inhibition. Our results support the idea that N/OFQ is an anti-reward peptide which can act specifically in the LH/PFA to regulate feeding.

## Materials and Methods

All experiments were performed following the Canadian Council on Animal Care Guidelines and as approved by the Memorial University Institutional Animal Care Committee. Sprague Dawley rats were obtained from the breeding colony at Memorial University. Animals were housed in a light and temperature controlled room (L:D = 12:12 h, lights on at 7:00 am, room temperature 22±1°C).

### Surgery and Behavioural Testing

Male-Sprague Dawley rats (250–300 g) were anesthetized with isoflurane (4% induction, 2% maintenance). A stainless-steel guide cannula (22-gauge; 0.028 in. OD, 0.022 in. ID) was implanted to the right LH/PFA using the following coordinates: 2.9 mm posterior and 1.2 mm lateral with respect to the bregma and 2.5 mm dorsal to the interaural line. Cannulae were fixed in place with skull screws and dental cement. Animals were housed individually and given a minimum of 5 days to recover from surgery before experimentation. All rats had *ad libitum* access to standard lab chow with the exception of testing days. Water was available at all times.

On test days, chow was removed at 9:00 am in order to prevent spontaneous food intake immediately prior to the test period. At 1:00 pm, rats were given free access to sucrose pellets (dustless precision pellets, F0021, Bio-Serv). Immediately preceding the presentation of the food, an injection cannula was inserted into the pre-implanted guide cannula to inject either N/OFQ (10 nmol in 0.5 µl) or vehicle (0.5 µl, control) into the LH/PFA over 60 sec, which was then left in place for an additional 30–60 sec to ensure proper diffusion at the injection site. N/OFQ was dissolved in sterile saline at the final concentration, made into aliquots and frozen at −20°C until use. The amount of food intake was measured at one and three hours post-injection. Chow was returned to the animals following the experiments. Each rat received paired injections, i.e. vehicle or drug application on two experimental days separated by a non-experimental day. The order of injections was alternated from animal to animal. The effect of N/OFQ was analyzed relative to the response to vehicle to account for variations among individual animals. In a separate cohort of rats, the same protocol was followed except instead of sugar pellets, intake of chow following vehicle or N/OFQ injection was examined.

After the food intake experiments, animals were euthanized with CO_2_ and 4% pontamine sky blue (0.5 µl) was injected via the injection cannula to aid in the localization of cannula placement. Brains were removed, fixed in 10% formalin overnight, cryoprotected in 20% sucrose, frozen and cut at 40 µm on a cryostat. Cannula placements were determined under a light microscope by two experimenters. Injections that fell completely within the area where orexin and MCH neurons are concentrated (based on [21]) were considered successful (on-target), whereas those with the injection site completely outside of the field were considered as off-target and served as a control group. Those with cannula placements at the borderline of the orexin/MCH field were excluded from the analysis.

### In vitro Electrophysiology

Rats (60–70 g) were deeply anesthetised with halothane, decapitated and brains were quickly removed. 250 µm-thick coronal hypothalamic slices were generated in ice-cold artificial cerebrospinal fluid (ACSF) using a vibratome (VT-1000, Leica Microsystems). The composition of the ACSF was as follows (in mM): 126 NaCl, 2.5 KCl, 1.2 NaH_2_PO_4_, 1.2 MgCl_2_, 25 NaHCO_3_, 2 CaCl_2_, 10 glucose, pH 7.3–7.35. Following dissection, slices were incubated in ACSF at 32–35°C for 30–45 min, then at room temperature until recording. ACSF was continuously bubbled with O_2_ (95%)/CO_2_ (5%).

Conventional whole-cell patch-clamp recordings were performed on brain slices perfused with ACSF at 1.5–2 ml/min, 26±1°C, using a Multiclamp 700B amplifier and pClamp 9.2 software (Molecular Devices, Sunnyvale, CA). Slices were visualized with a microscope equipped with infrared-differential interference contrast optics (DM-LFSA, Leica Microsystems), to target neurons with large cell-bodies (>15 µm diameter) and located within the LH/PFA dorsal to the fornix. To increase the extracellular K^+^ concentration to 10 mM, 7.5 mM of NaCl in ACSF was replaced by KCl. Pipette resistance when filled with internal solution ranged from 3 to 7 MΩ. Three different conventional whole cell internal solutions were used in the present study. The first one contained (in mM): 123 K-gluconate, 2 MgCl_2_, 8 KCl, 0.2 EGTA, 10 HEPES, 4 Na_2_-ATP, 0.3 Na-GTP. For the high-chloride internal solution, K-gluconate was replaced with equimolar KCl. Another conventional whole cell solution used in the present study consisted of (in mM) 120 K-gluconate, 1 NaCl, 1 MgCl_2_, 1 CaCl_2_, 10 HEPES, 10 EGTA, 3 K_2_ATP. For perforated patch recordings, nystatin (final concentration 450 µg/ml) and pluronic acid were dissolved in DMSO and added to an internal solution that contained the following: 120 K-gluconate, 5 MgCl2, 10 EGTA, 40 HEPES. KOH was added to all internal solutions until pH 7.3–7.35 was reached. Upon attaining whole cell access (series resistance 10–25 MΩ), each neuron’s electrophysiological characteristics were observed by applying a series of 300 msec hyperpolarizing (−200 and −100 pA) and depolarizing (100 and 200 pA) current injections in current clamp mode. Voltage responses typical of orexin neurons included a lack of spike adaptation upon positive current injection, the presence of spontaneous action potentials, I*_h_* and rebound depolarization upon relief from hyperpolarizing steps [22]. Only cells displaying these electrophysiological characteristics were included in the present study. Voltage clamp experiments were performed at a holding potential of −70 mV with the exception of voltage ramps, in which the membrane potential was ramped from −140 to −20 mV in 600 msec. Miniature excitatory postsynaptic currents (mEPSCs) were recorded with tetrodotoxin (TTX, 1 µM) and picrotoxin (50 µM) added to the ACSF. Series resistance was monitored throughout the experiment by regularly applying 100 msec, −20 mV square pulses. The resulting transient currents were blanked in the representative voltage clamp traces in figures for clarity of presentation. Cells in which the series resistance changed by >20% during the course of the experiment were not included for analysis.

All compounds were applied through the bath perfusion at the final concentrations. TTX was obtained from Alomone Labs (Jerusalem, Israel), picrotoxin and GDPβS were obtained from Sigma-Aldrich (St. Louis, MO). N/OFQ (1–13)NH_2_, UFP-101, tertiapin Q and glibenclamide were obtained from Tocris Bioscience (Ellisville, MO). N/OFQ (1–13)NH_2_ is a bioactive metabolite of N/OFQ and was used in the present study as a NOP receptor agonist. N/OFQ (1–13)NH_2_ is referred to as N/OFQ for the remainder of the Methods and throughout the Results section. Antagonists and channel inhibitors were applied for a minimum of 5 min before N/OFQ was tested. For kinase inhibitor experiments, slices were incubated for more than an hour before being transferred into the recording chamber, where they were continuously perfused with the same inhibitor throughout the experiment.

**Figure 1:**
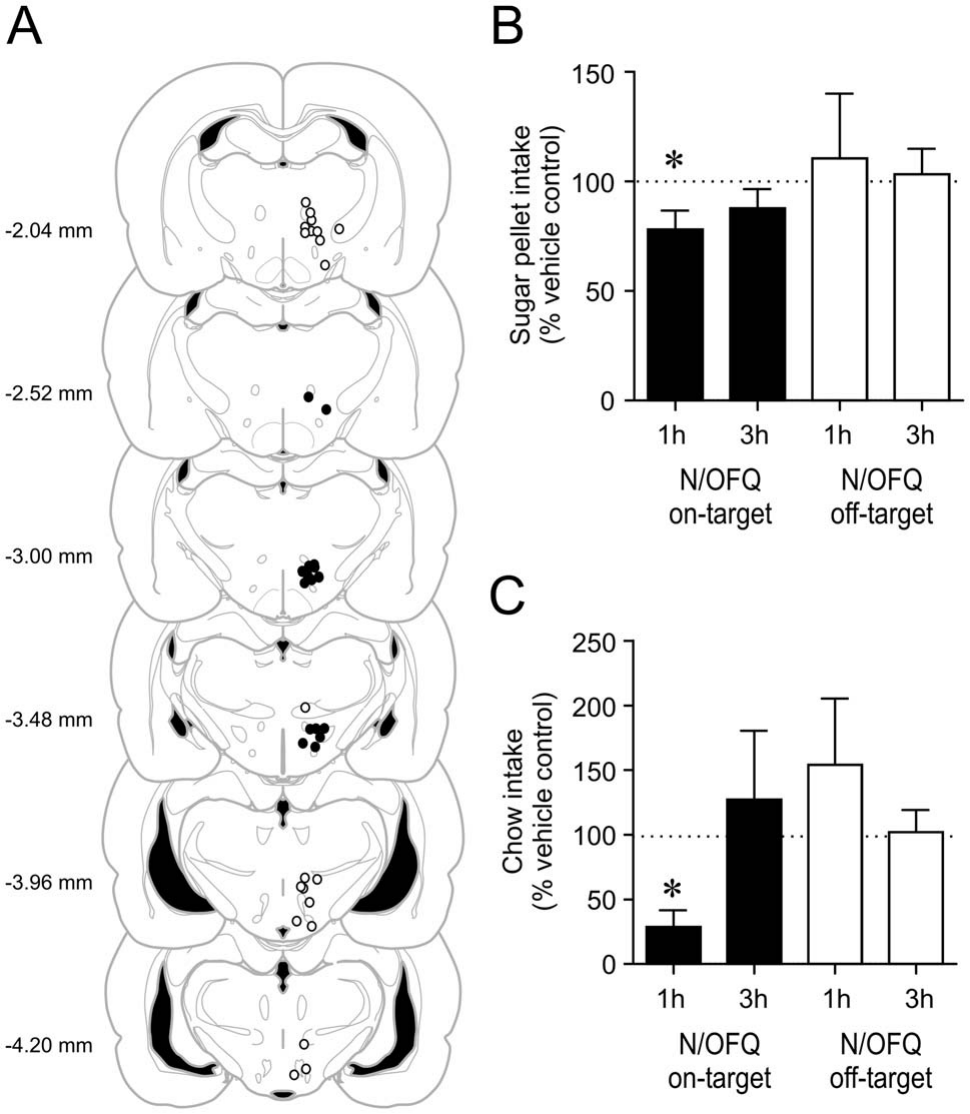
Intra-LH/PFA N/OFQ injection decreases palatable food intake. *A*: Coronal brain maps depicting the location of injections. Each circle represents a rat used for the analysis. Filled and open circles were categorized as on- or off-target, respectively, relative to the orexin/MCH field. Numbers on the left of each map indicate the posterior distance from the bregma based on the Rat Brain Atlas by Paxinos and Watson [57]. The brain maps were modified from the images in the Atlas. *B*: N/OFQ significantly reduced the sugar pellet intake during the first hour (1 h) following an injection into the LH/PFA (on-target) but not the 3h-total intake post-injection (filled bars). In contrast, N/OFQ injections outside of the LH/PFA (off-target) did not affect the sugar pellet consumption (open bars). *C*: N/OFQ significantly reduced chow intake during the first hour (1 h) following an injection into the LH/PFA (on-target) but not the 3h-total intake (filled bars). This effect was not seen when the injection was off-target. * p<0.05.

### Post-Hoc Immunohistochemistry

Biocytin (1–1.5 mg/ml) was added to internal solutions for a subset of recordings to label cells for post-hoc immunohistochemical phenotyping. Immediately following recording, sections were fixed in 10% formalin for >16 hours, washed in PBS then treated with a cocktail of goat anti-orexin A (1∶1000–3000; Santa Cruz Biotechnology, Santa Cruz, CA, USA) and rabbit anti-MCH (1∶1000–4000; Phoenix Pharmaceuticals, Belmont, CA, USA) antibodies for 3 days at 4°C, followed by a cocktail of Cy3-conjugated donkey anti-goat, Cy2-conjugated donkey anti-rabbit and streptavidin-conjugated AMCA (1∶500; Jackson ImmunoResearch, West Grove, PA, USA). Stained sections were visualized using a fluorescence microscope to detect MCH (Cy2), Orexin-A (Cy3) and biocytin (AMCA). Among 37 neurons with the electrophysiological profile typical of orexin neurons and filled with biocytin, none were MCH-immunopositive, 31 were confirmed to be orexin-A immunopositive, while 6 were negative for orexin A (success rate of identifying orexin neurons 31/37 = 83.8%). These orexin A-negative cells were excluded from analysis. Some of the immunonegative results may be explained by technical difficulty associated with staining thick sections. The high success rate agrees with previous work from our lab [23] that orexin neurons can be reliably identified based on electrophysiological characteristics. Thus, as stated earlier, anatomical and electrophysiological features were used as the primary means to distinguish orexin neurons for this study.

**Figure 2:**
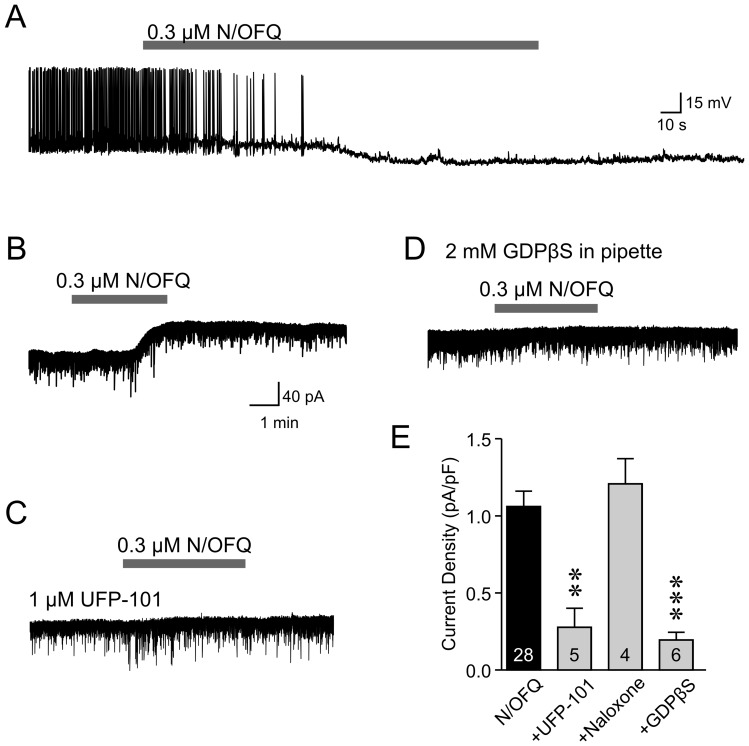
N/OFQ inhibits orexin neurons via NOP receptor activation in the rat. *A*: N/OFQ hyperpolarizes orexin neurons. *B*: Representative voltage clamp trace showing a TTX-insensitive outward current induced by N/OFQ application. *C*: N/OFQ-induced effect is significantly attenuated by UFP-101. However, the effect persists in the presence of naloxone (E), indicating a specific activation of NOP receptors. D: N/OFQ is ineffective on orexin neurons patched with GDPβS in the recording pipette. E: Grouped data is shown. Numbers in bars represent the number of cells examined in each group. ** p<0.01, *** p<0.005 vs. N/OFQ.

### Data Analysis

Membrane potential and holding current were measured using Clampfit 9.2 (Molecular Devices; Sunnyvale, CA) and miniature excitatory postsynaptic potentials (mEPSCs) were analyzed using MiniAnalysis (Synaptosoft; Decatur, GA). Current density was calculated by dividing the measured current (pA) by the membrane capacitance (pF). The statistical tests used included one-way ANOVA with Dunnett’s post test for multiple group comparisons and two-tailed paired and unpaired Student t-tests for two-group comparisons. Kolmogorov-Smirnov tests were used for mEPSC analysis. Data are expressed as mean ± S.E.M. p<0.05 was considered significant.

**Figure 3:**
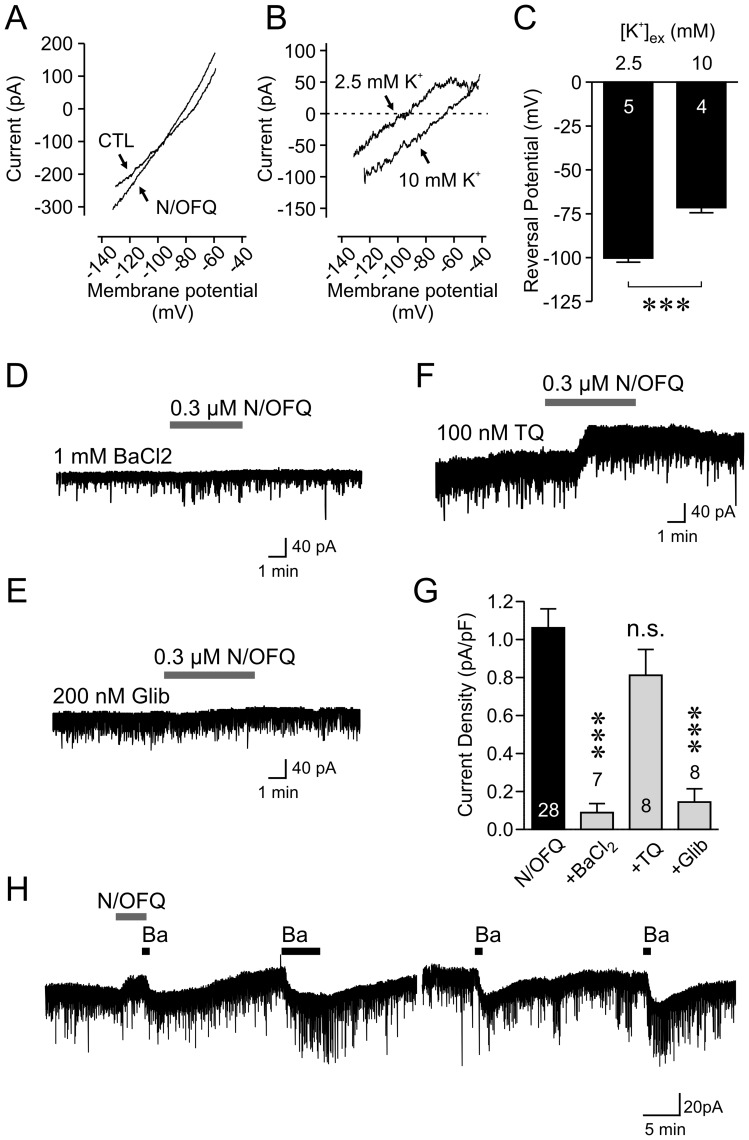
N/OFQ activates KATP channels in orexin neurons. *A*: Current responses to voltage-ramps before (control; CTL) and during N/OFQ application in 2.5 mM extracellular potassium. B: Subtraction of currents recorded in two conditions (N/OFQ - CTL) reveals an N/OFQ-induced current (2.5 mM K^+^). Increasing the extracellular potassium concentration to 10 mM shifted the current-voltage relationship rightward. *C*. Grouped data showing that the reversal potential of the N/OFQ current is dependent on the concentration of extracellular potassium. *** p<0.005. *D-F*: N/OFQ responses are attenuated by BaCl_2_ (*D*) and glibenclamide (Glib) (*E*) but not tertiapin Q (TQ) (*F*). Grouped data are shown in (G). *** p<0.005, n.s. non-significant vs. N/OFQ. Numbers in bars represent the number of cells examined in each group. *H*: Once N/OFQ current is induced, it is persistent and retains Ba^++^ sensitivity even after a prolonged washout.

## Results

### Effect of Intra LH/PFA N/OFQ on Food Intake

To determine a site-specific role of N/OFQ, we investigated how the intake of sugar pellets is affected by a local N/OFQ administration into the LH/PFA. When N/OFQ was injected at a dose (10 nmol) shown to increase feeding in the ventromedial nucleus or nucleus accumbens [13], this resulted in a significant reduction in the sugar intake during the first hour of feeding compared to vehicle injection (Fig. 1B, n = 10, p<0.05, paired t-test). By the 3^rd^ hour, the difference in pellet intake disappeared as a group (n = 10, p>0.05, paired t-test). Nonetheless, 7 out of 10 animals tested consumed less (more than 20% reduction) during the first 3 hours following N/OFQ compared to vehicle injection. When the cannula placement was outside of the LH/PFA (off-target), N/OFQ did not have any significant effect (n = 11, p>0.05 for 1 h and 3 h post-injection, paired t-test). We also found that in rats that received N/OFQ into the LH/PFA (on-target), chow intake during the first hour post-injection was significantly reduced compared to that after saline (Fig. 1C, n = 4, p<0.05, paired t-test), while the difference became non-significant after 3 hours (p>0.05, paired t-test). Again, N/OFQ had no effect on the chow intake in animals whose injection site was outside of the LH/PFA (off-target) (Fig. 1C, n = 10, p>0.05 for 1 h and 3 h post-injection). These results suggest that the local action of N/OFQ in the LH/PFA is to inhibit food intake in general.

**Figure 4:**
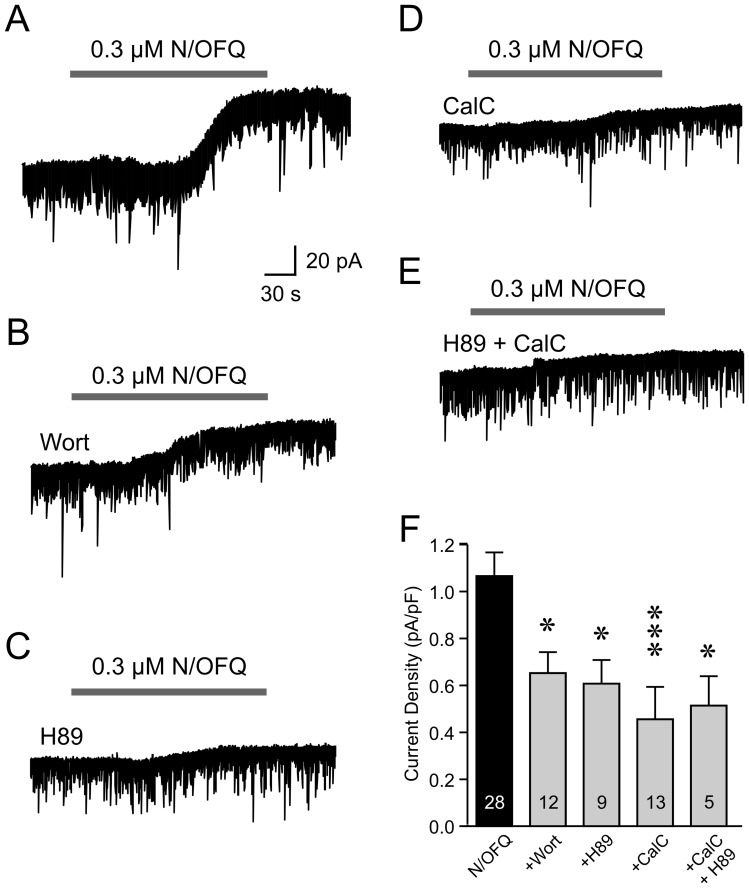
N/OFQ-induced KATP channel activation is mediated by PI3K, PKA and PKC. *A-E*: Representative recordings showing the effect of N/OFQ in the presence of specific inhibitors for PI3K (wortmannin; Wort), PKA (H89) or PKC (calphostin C; CalC), as indicated. Scale bars in *A* apply to all traces. *F*: Grouped data showing that kinase inhibitors attenuate the N/OFQ effect. Combined application of H89 and CalC did not have an additive effect. Numbers in bars represent the number of cells examined in each group. * p<0.05, *** p<0.001 vs. N/OFQ.

### N/OFQ Effect on Rat Orexin Neurons

Next, we used patch clamp recordings to determine the cellular mechanism underlying the N/OFQ effect on orexin neurons. We found that bath application (2–5 min) of N/OFQ (300 nM) induced a robust and long-lasting hyperpolarization in rat orexin neurons (-15.6±0.7 mV, n = 4, Fig. 2A), in agreement with a previous study in mice [20]. This concentration was used for the remaining electrophysiological experiments. In voltage clamp mode, the N/OFQ effect was detected as an outward current in all orexin neurons tested (n = 28, Fig. 2B, E) using four different internal solutions (K gluconate-based with Na_2_-ATP, n = 16; K gluconate-based with K_2_-ATP, n = 5; KCl-based, n = 3; nystatin-perforated patch, n = 4). The input resistance for these cells was 518±41 MΩ, which falls within the range of values previously reported for the same measure in mouse orexin neurons [24]–[27]. The amplitude of the outward currents was similar regardless of the internal solution used (p>0.05, one-way ANOVA), and thus data were grouped together. The effect was TTX-insensitive (n = 8 with 1 µM TTX, n = 20 without TTX; p>0.05, unpaired t-test) and abolished by the selective competitive NOP receptor antagonist UFP-101 (1 µM; Fig. 2C, E, n = 5, p<0.01, Dunnett’s test) but not naloxone, the antagonist for the classic opioid receptors (10 µM; Fig. 2E, n = 4, p>0.05, Dunnett’s test). The N/OFQ current was also significantly attenuated by postsynaptic loading of the non-hydrolysable GDP analogue GDPβS (2 mM; Fig. 2D, E, n = 6, p<0.005, Dunnett’s test). These results suggest that N/OFQ directly activates G-protein coupled NOP receptors on orexin neurons.

**Figure 5:**
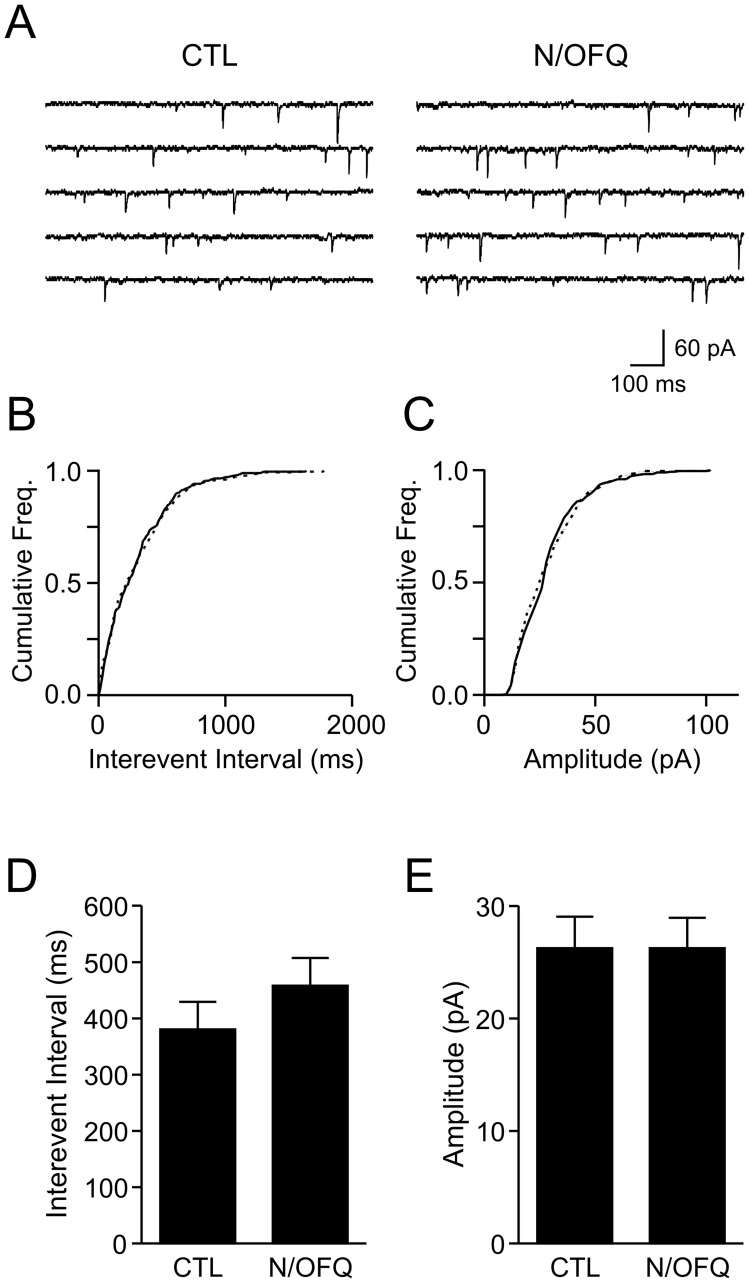
N/OFQ does not consistently modulate excitatory transmission to orexin neurons. *A*: mEPSC traces from a representative orexin neuron before (control: CTL) and during N/OFQ (0.3 µM, 5 min) application, showing a lack of effect. *B-C*: Cumulative frequency (freq.) graphs for mEPSC interevent interval (*C*) and amplitude (*D*) for the cell shown in *A*. Solid and broken lines represent CTL and N/OFQ conditions, respectively. *D-E*: Grouped data (n = 8) of mEPSC interevent interval (*D*) and amplitude (*E*) in control and N/OFQ.

Using a voltage ramp protocol, the reversal potential of the N/OFQ-induced current was examined, which was near the theoretical potassium equilibrium potential of −102.1 mV (n = 5, Fig. 3A), again consistent with the mouse study [20]. We further confirmed this to be potassium current by increasing the extracellular potassium concentration by four-fold, which depolarized the reversal potential close to its theoretical value of −66.3 mV (n = 4, Fig. 3A–C, p<0.0001 vs. standard ACSF, unpaired t-test). The lack of effect of the internal solution used on the magnitude of the N/OFQ current is consistent with the small variability of the potassium equilibrium potential for each of the different internal solutions (range from −99.8 to −102.1 mV). We also found that the N/OFQ response was inhibited by 1 mM BaCl_2_ (n = 7, Fig. 3D and G, p<0.005, Dunnett’s test), suggesting the involvement of barium-sensitive inwardly-rectifying potassium channels. These include GIRK and ATP-sensitive potassium (KATP) channels, which can be blocked using tertiapin Q and glibenclamide, respectively. Blocking KATP channels with 200 nM glibenclamide abolished the N/OFQ response (Fig. 3E and G, n = 8, p<0.005 vs. N/OFQ alone, Dunnett’s test). In contrast, in the presence of tertiapin Q at a concentration shown to attenuate the N/OFQ-induced effect in MCH neurons (100 nM) [19], N/OFQ was still capable of inducing an outward current (Fig. 3F and G, n = 8, p>0.05 vs. N/OFQ alone, Dunnett’s test). The N/OFQ current was persistent and did not return to baseline even after an hour following agonist washout, but remained sensitive to barium (Fig. 3H). Taken together, the inhibitory effect of N/OFQ is mediated by prolonged activation of KATP channels in orexin neurons.

KATP channels have been shown to be regulated by a variety of protein kinases, including phosphatidylinositol 3-kinase (PI3K) [28], protein kinase A (PKA) [29] and protein kinase C (PKC) [30]. To determine the intracellular mediator(s) of N/OFQ-induced KATP channel activation, we tested the effect of N/OFQ on orexin neurons treated with wortmannin (Wort; 500 nM), H89 (10 µM) or calphostin C (CalC; 100 nM), inhibitors of PI3K, PKA and PKC, respectively. As shown in Figure 4, we found that all three kinase inhibitors partially but significantly attenuated the effect of N/OFQ (p<0.001, one-way ANOVA). Co-treatment with CalC and H89 suppressed the N/OFQ current (n = 5, p<0.05 vs. N/OFQ alone, Dunnett’s test) but did not show an additive effect. These results suggest that PI3K, PKA and PKC play a role in the activation of KATP channels by N/OFQ.

To determine whether N/OFQ has an additional effect on the excitatory synaptic transmission, we examined its effect on mEPSCs. We found that in 2 of the 8 cells examined, there was a significant and reversible decrease in mEPSC inter-event interval (p<0.05, Kolmogorov–Smirnov test) without a change in amplitude (p>0.05, Kolmogorov–Smirnov test). However, as a group, N/OFQ affected neither the interevent interval (n = 8, p>0.05, paired t-test) nor the amplitude of mEPSCs (n = 8, p>0.05, paired t-test) (Fig. 5). Thus, in the rat, the presynaptic modulation by N/OFQ only occurs in a minor subpopulation of orexin neurons.

## Discussion

Previous studies have shown that N/OFQ inhibits orexin neurons in vivo [31] and in vitro [20]. Here, we have extended these findings to show that N/OFQ hyperpolarizes orexin neurons by inducing a prolonged activation of KATP channels which is mediated by PKA, PKC and PI3K. In contrast to the previous report by Xie et al. [20], we found no effect of N/OFQ on excitatory synaptic transmission in the majority of orexin neurons tested. This may be due to differences in animal models as Xie and colleagues utilized orexin/EGFP and orexin/YC2.1 transgenic mice of both sexes while we used male rats. The composition of internal solutions also differed but this is unlikely to account for the discrepancy as the reported effect on excitatory transmission is presynaptic, not postsynaptic [20]. Nonetheless, our work confirms a robust and direct inhibitory effect which solidifies N/OFQ as a negative regulator of orexin neurons, which may be involved in the hypophagic action of N/OFQ in the LH/PFA.

### N/OFQ Activates KATP Channels in Orexin Neurons

N/OFQ activates inwardly rectifying potassium channels in a number of neuronal populations, some of which have demonstrated barium sensitivity [32]–[36]. These characteristics have been occasionally interpreted as GIRK currents since NOP receptors are G protein coupled. However, the availability of specific GIRK and KATP channel blockers allowed us to determine that NOP receptor activation results in KATP currents in orexin neurons (current study) and GIRK currents in MCH neurons [19], both of which are barium sensitive. Therefore, the barium sensitivity and inward rectification without the use of specific inhibitors are insufficient to demonstrate GIRK channel activation.

Our data suggest that in orexin neurons, NOP receptors partially signal via PI3K, PKA and PKC, as has been shown in other cell types [37]–[40]. Furthermore, each of these kinases has been implicated in the regulation of KATP currents [28]–[30], [41]. It appears that there is some overlap in the signaling by these kinases as the average inhibition of N/OFQ-induced currents by wortmannin, H89 and calphostin C was 38.1, 44.5 and 58.5% of control, respectively, which add up to be 141.1%. Should they act independently, the added figure should not exceed 100%. Furthermore, co-application of PKA and PKC inhibitors did not induce an additive effect. This result indicates that PKA and PKC signaling may converge onto a common signaling molecule such as mitogen-activated protein kinase [42], which in turn can activate KATP channels [43]. Alternatively, PKA and PKC may comprise a single sequential signaling pathway, for example by PKA activating PI3K [44] followed by lipid products of PI3K activating PKC [45]. PI3K can also be activated independently by NOP receptors since PI3Kγ, a member of class 1B PI3K, is a major effector of Gβγ subunits {Leopoldt, 1998 2117/id}.

The source of endogenous N/OFQ released in the LH/PFA remains unknown, since N/OFQ is expressed in a wide range of brain areas [47] including those that send projections to the orexin field [48]. Many N/OFQ-immunopositive fibers are found in the hypothalamus, some of which make apparent synaptic contacts with orexin neurons [20], [31]. It is likely that some of these fibers originate from orexin neurons as they co-express N/OFQ [49] and synapse onto one another [50]. Our study showing a persistent effect of N/OFQ which outlasts prolonged agonist washout suggests that N/OFQ provides a tonic inhibitory tone that may be important for preventing overexcitation from converging excitatory inputs that stimulate orexin neurons [51]. The time course of the N/OFQ effect adds another layer of complexity associated with cotransmission of orexin and opioids (dynorphin and N/OFQ) by orexin neurons, since the balance between excitatory and inhibitory actions of orexin and dynorphin, which have different desensitization rates, has been shown to shift with time [52].

### Physiological Significance

N/OFQ has been known to elicit feeding when injected into a number of brain sites, including the lateral ventricles, ventromedial and arcuate hypothalamic nuclei and nucleus accumbens shell [11]–[13]. In contrast, we found that the local injection of N/OFQ into the LH/PFA decreased the intake of sugar pellets and chow. The LH/PFA is populated by orexin and MCH neurons known to promote the consumption of drugs of abuse [15], [16], palatable food [17], [18], [53] as well as regular lab chow [17], [18], [53], [54]. This, together with the known anti-reward properties of N/OFQ, suggests that orexin and MCH neurons mediate the inhibitory effect of N/OFQ on the motivational aspect of food intake.

N/OFQ acts as a functional “anti-opioid peptide” in nociception [55], motor activity [56] and reward [7], [8]. In addition, the results of the present study extend this to include food intake. N/OFQ’s inhibitory effects on stress-induced analgesia and food intake can be attributed, at least partially, to its effect on orexin neurons ([20], [31] and present study). It remains to be seen whether these functions are mediated by the same or distinct subgroups of orexin neurons. Nonetheless, we found all orexin neurons tested to invariably respond to N/OFQ with an outward current or hyperpolarization, which was consistently abolished by KATP channel blockers. This suggests that the sensitivity to N/OFQ is a common feature of this peptidergic cell type and that the same cellular mechanism may be involved in the analgesic and feeding effects. Therefore, N/OFQ is an important modulator of the orexin system.
